# Expression profile of inflammatory cytokines in aqueous from glaucomatous eyes

**Published:** 2012-02-11

**Authors:** Jocelyn Chua, Maya Vania, Chui Ming Gemmy Cheung, Marcus Ang, Soon Phaik Chee, Henry Yang, Jing Li, Tina T. Wong

**Affiliations:** 1 Singapore National Eye Centre, Singapore; 2 Singapore Eye Research Institute, Singapore; 3 Singapore Cancer Institute, Singapore; 4 Department of Ophthalmology, Yong Loo Lin School of Medicine, National University of Singapore, Singapore; 5 Department of Ophthalmology, Xin Hua Hospital, Shanghai Jiao Tong University School of Medicine, Shanghai, China; 6 Materials Science and Engineering, Nanyang Technological University, Singapore

## Abstract

**Purpose:**

To determine the proinflammatory cytokine profile of aqueous humor from glaucomatous eyes.

**Methods:**

Aqueous humor samples were prospectively collected from 38 eyes (26 primary open angle glaucoma [POAG] and 12 primary angle closure glaucoma [PACG] eyes) of 37 medically treated glaucoma patients and 23 cataract subjects recruited in an institutional setting in this case-controlled study. The main outcome measure was to quantify the levels of 29 inflammatory cytokines in the aqueous of glaucoma and cataract subjects using a multiplexed cytokine analysis. Data on patient demographics, duration of glaucoma, preoperative intraocular pressure (IOP) as well as duration of anti-glaucoma therapy were also collected for correlation analysis.

**Results:**

Mean duration of glaucoma was 53.8 months (range 1–360 months). Aqueous obtained from the glaucoma patients showed increased concentration of interleukin (IL)-9 (p=0.032), IL-12 (p=0.003), interferon (IFN)-α (p=0.034), IFN-γ (p=0.002), monokine induced by interferon-gamma (MIG or CXCL9) (p=0.006), and IL-10 (p=0.050), compared to the cataract group. The POAG group had higher IL-12 (p=0.011), IFN-γ (p=0.005), and CXCL9 (p=0.047) levels than controls, while the PACG group had higher interleukin-8 (CXCL8) (p=0.015) and CXCL9 (p=0.023) levels than the controls. No significant correlation was observed between aqueous cytokine level and preoperative IOP and duration of glaucoma. Duration of topical Timolol and Alphagan therapy correlated negatively with CXCL8 (r=-0.588, p=0.035), respectively.

**Conclusions:**

Primary glaucoma is associated with an aqueous inflammatory response and this is different between POAG and PACG groups. Duration of glaucoma treatment may have an effect on cytokine profile in the aqueous.

## Introduction

Glaucoma is characterized by the presence of a typical glaucomatous optic neuropathy with a corresponding visual field defect and is usually associated with an elevated intraocular pressure (IOP), which is a known risk factor for disease progression [1,2]. In glaucoma, aqueous humor drainage through the trabecular meshwork largely determines the IOP and an elevated IOP is a result of trabecular meshwork resistance to aqueous outflow. The aqueous humor is important for the maintenance of both physiologic function and metabolic homeostasis of intraocular structures within the anterior chamber such as the trabecular meshwork, iris, corneal endothelium, and lens [3].

Altered cytokine and chemokine composition of the aqueous humor has been observed in various ocular disease conditions including uveitis, diabetic retinopathy and age-related macular degeneration [4,5]. Increased concentrations of tumor necrosis factor (TNF)-α [6], transforming growth factor (TGF)-β2 [7-9], vascular endothelial growth factor (VEGF) [10], interleukin (IL)-6 [11,12],and interleukin-8 (CXCL8) [11,13] have been observed in glaucomatous aqueous humor. The significance and role in which these aqueous cytokines may play in the pathogenesis of primary glaucoma is still unclear. Age-related accumulation of oxygen free radicals can lead to the induction of oxidative stress related trabecular cell death as well as the upregulation of several inflammatory cytokines such as TGF-β2, IL-1, IL-6,and CXCL8 [14,15]. Other cytokines have also been proposed to contribute to changes in expression levels of matrix metalloproteinase (MMP) and tissue inhibitors of metalloproteinase (TIMP) [16] in the trabecular meshwork resulting in an alteration in extra-cellular matrix production that can give rise to an increase in aqueous outflow resistance.

To date, the profile of aqueous cytokines in the two primary glaucoma subgroups is still not fully described. This study reports the concentrations of 29 aqueous cytokines in patients with primary open angle glaucoma (POAG) and primary angle closure glaucoma (PACG) in an Asian population. Correlations of elevated aqueous cytokines to demographic and clinical factors are also reported.

## Methods

### Patient eligibility and recruitment

This study was approved prospectively by the local Institutional Review Board (IRB) at the Singapore National Eye Centre and performed in accordance to the tenets of the Declaration of Helsinki. Thirty-seven glaucoma patients on topical medication and 23 non-glaucomatous cataract patients with cataract and no previous history of any topical medical therapy were recruited from the glaucoma subspecialty clinic and general comprehensive clinic respectively at the Singapore National Eye Centre. Any patient with a known systemic inflammatory, autoimmune or immunosuppressive disease as well as a pre-existing ocular disease (retinal vein occlusion, retinal artery occlusion, diabetic retinopathy, age-related macular degeneration) or previous ocular surgery was excluded from the study. Informed written consent with regard to donation and storage of human fluid samples was obtained from all patients.

Of the 38 eyes of 37 glaucoma patients, 26 had a diagnosis of primary open angle glaucoma (POAG) and 12 with primary angle closure glaucoma (PACG). The diagnosis of primary glaucoma and its subtypes was based on the IOP, glaucomatous nerve damage (defined by the presence of neuro-retinal rim loss of the optic nerve head with a vertical cup-disc ratio of 0.7 or more) with corresponding visual field loss, presence of occludable angles on indentation gonioscopy (PACG is diagnosed when the pigmented trabecular meshwork was not visualized in at least 2 quadrants with or without peripheral anterior synechiae) and the absence of features suggestive of a secondary etiology. Only 1 of 12 PACG eyes had a prior history of acute angle closure. Laser peripheral iridotomy was performed in all PACG eyes upon diagnosis, followed by a topical steroid therapy for a period of 2 weeks. Trabeculectomy with mitomycin C was performed in glaucomatous eyes with sub-optimally medically controlled IOP as well as progressive visual field loss and optic disc cupping; and in the presence of a visually significant cataract, a combined phacotrabeculectomy surgery was then performed. Prior to surgery, 34 glaucoma eyes were treated with a beta-blocker Timolol 0.5% (Timoptic; Merck, Whitehouse Station, NJ), 28 eyes with a prostaglandin analog (PGA; Travoprost: Travatan, Alcon, Fort Worth, TX; Latanoprost; Xalatan; Pfizer, New York, NY; Bimatoprost; Lumigan; Allergan Inc., Irvine, CA), 12 eyes with an alpha-agonist Brimonidine (Alphagan; Allergan Inc.) and 8 eyes with a carbonic anhydrase inhibitor (CAI; Brinzolamide: Azopt; Alcon; Dorzolamide: Trusopt; Merck). Six eyes were on monotherapy while the rest were on at least 2 eye drops. Routine phacoemulsification surgery with intraocular lens implant was performed for all 23 non-glaucomatous eyes with visually significant cataract.

### Aqueous collection

Aqueous humor collection was performed under sterile conditions before commencement of surgery for all patients. In all PACG eyes, aqueous samples were collected at least 1 month after laser peripheral iridotomy procedure. In the only eye with a previous acute angle closure, aqueous was collected one month after the acute attack has resolved. One-hundred micro liters (100 μl) of aqueous humor was withdrawn using a 30-gauge needle on a tuberculin syringe via an anterior chamber paracentesis. Aqueous sample was frozen at −80 °C within 2 h of collection till further analysis.

### Cytokine analysis

Bio-Plex Pro^TM^ magnetic color-bead-based multiplex assay (Bio-Rad Laboratories, Inc., Hercules, CA) was used to measure the concentrations of the following human cytokines/chemokines: IL-1Ra (Interleukin-1 receptor antagonist), IL-1β, IL-2, IL-4, IL-5, IL-6, IL-7, IL-9, IL-10, IL-12, IL-13, IL-15, IL-17, G-CSF (granulocyte colony-stimulating factor), GM-CSF (granulocyte- macrophage colony-stimulating factor), IFN-γ (interferon-gamma), CCL2 (MCP-1, monocyte chemotactic protein-1), CCL3 (MIP-1α, macrophage inflammatory protein-1-alpha), CCL4 (MIP-1β, macrophage inflammatory protein-1-beta), CCL5 (RANTES, Regulated upon Activation, Normal T-cell Expressed, and Secreted), CCL11 (Eotaxin, eosinophil chemotactic protein), CXCL8 (IL-8), CXCL9 (MIG, monokine induced by interferon-gamma), CXCL10 (IP-10, interferon-gamma-induced protein 10), TNF-α (tumor necrosis factor-alpha), IFN-α (interferon-alpha), PDGF-BB (platelet-derived growth factor-BB), basic-FGF (basic fibroblast growth factor) and VEGF (vascular endothelial growth factor). The assay was conducted according to the manufacturer’s instruction. Thirty-five micro liters (35 μl) of aqueous humor sample was used in each reaction. Fluorescence intensity (FI) from the immunoassay was acquired and analyzed using Bio-Plex Manager 6.0. Concentrations that were lower than the low limit of detection (LOD) was defined as non-measurable.

### Classification analysis

Step-wise analysis was performed to generate cytokine profiles to best separate glaucoma aqueous samples from cataract samples and to separate POAG from PACG. For each cytokine, the measured concentration was logarithmically transformed. An initial analysis was used on all available cytokines, which generated a poor separation between groups. The analysis was then refined, by using cytokines and chemokines that showed significant differences between the compared groups by two-sample Student’s *t*-test (p-value ≤0.05). The panel of differentially expressed cytokines was then further reduced by the removal of highly correlated cytokines. The accuracy of the heat map separation for each group was calculated as follow: the number of true subjects (based on clinical diagnosis) divided by the number of total subjects within the group and the result multiplied by 100%. A separate decision tree analysis was performed to select step-wise marker cytokines using WEKA, a machine learning algorithms for data mining tasks. A fivefold cross validation was conducted to examine the error rate of this method.

### Statistical analysis

Statistical analysis was performed using Predictive Analytics Soft Ware (PASW) Statistics 18 (IBM Corporation, Armonk, NY). Statistical significance was accepted at p≤0.05. For categorical variables, the frequency distribution and percentages were calculated and compared by Pearson χ^2^ analysis. For numerical variables in parametric distribution, the one-way ANOVA analysis was performed. For differences in cytokine concentrations, the Mann–Whitney U test with Bonferroni correction was performed and the resulting p-values were reported. For comparisons involving the PACG group, a multivariate binary logistic regression analysis was also performed, to adjust for the differences in axial length and the resulting p-values were reported. The two-tailed, nonparametric Spearman method was used to assess for correlations between variables.

## Results

### Patient demographics

Mean age of the glaucoma group was 69.9 years (range 58.7–84.4) and that of the cataract group was 66.5 years (range 26.6–88.8; p=0.228) as shown in Table 1. The mean duration of glaucoma from diagnosis was 53.8 months (range 1–360 months). Phacotrabeculectomy and trabeculectomy surgery were performed for 19 and 4 eyes, respectively. The remaining 15 glaucoma eyes underwent phacoemulsification surgery alone. The mean axial length measurements of both the glaucoma and cataract groups were shown in Table 1. The PACG group had a significantly shorter axial length when compared to both POAG (p=0.029) and cataract groups (p=0.023). No statistically significant difference in axial length was observed between the POAG and cataract groups.

**Table 1:** Demographic and clinical data of glaucoma and cataract patients.

**Characteristics**	**Glaucoma patients**	**Cataract non-glaucomatous patients**	**p-value**
Total number	37	23	** **
Glaucoma subgroups	25 POAG	** **	** **
** **	12 PACG	** **	** **
Age (Mean±SD^a^)	69.9±7.6	66.5±11.8	0.228^d^
Gender	** **	** **	0.926^e^
Male	17	11	** **
Female	20	12	** **
Race	** **	** **	0.600^e^
Chinese	33	19	** **
Malay	3	2	** **
Indian	1	1	** **
Others	0	1	** **
AXL^b^ (Mean±SD)	POAG: 24.2±1.8	23.8±1.6	0.026^df^
	PACG: 23.0±0.9		0.250^dg^
** **	** **	** **	0.023^dh^
Surgery types	Number of eyes	Number of eyes	** **
Phacotrabeculectomy	19	0	** **
Trabeculectomy	4	0	** **
Phacoemulsification	15	23	** **
Medical Therapy^c^	Number of eyes	Number of eyes	** **
BB	34	0	** **
PGA	28	0	** **
AA	12	0	** **
CAI	8	0	** **
Number of drops			
One	6	0	** **
Two or more	32	0	** **

**^a^**SD: Standard deviation; **^c^**AXL: axial length; **^c^**BB: beta-blocker, AA: alpha-agonist, CAI: carbonic anhydrase inhibitor, PGA: prostaglandin analog; **^d^**2-samples independent *t*-test; **^e^**Pearson's χ^2^; **^f^**between POAG and PACG; **^g^**between POAG and cataract; **^h^**between PACG and cataract.

### Aqueous cytokines

The aqueous concentrations of 29 cytokines in both the glaucoma and cataract groups were measured. The aqueous cytokines were compared between groups in two aspects: the percentage of samples with measurable concentrations and the median concentration for each cytokine.

IL-4, IL-5, CCL3, and G-CSF was detected in less than 50% of samples in both control and glaucoma groups and therefore were not included in further analysis. The concentrations as well as the sensitivity of analysis for the rest of the cytokines are shown in Table 2.

**Table 2:** Aqueous cytokine concentrations in glaucoma and non-glaucomatous cataract groups.

** **	**Cataract (n=23)**			**Glaucoma (n=38)**			** **	** **
**Cytokines**	**Median**	**Range**	**Measurable**	**Median**	**Range**	**Measurable**	**p-value^a^**	**Sensitivity**
IL-2*	2.16	0–8	14/23	3.01	0–27	34/38	0.154	1.6
IFN-γ*	12.73	0–91	12/23	26.98	0–1167	36/38	**0.002**	6.4
IL-13	3.85	0–8	19/23	4.82	0–46	35/38	0.432	0.7
IL-17	2.27	0–11	8/23	3.98	0–241	22/38	0.227	3.3
IL-6*	2.23	0–14	9/23	4.02	0–89	27/38	0.789	2.6
IL-10*	1.45	0–5	18/23	1.91	0–64	37/38	**0.050**	0.3
IL-12*	7.15	0–27	17/23	14.06	0–68	36/38	**0.003**	3.5
IL-1β*	0.60	0–6	14/23	0.72	0–5	33/38	1.000	0.6
TNF-α*	9.58	0–34	16/23	12.65	0–82	35/38	0.086	6.0
IL-1Ra	8.58	3–96	10/17	8.74	0–84	14/34	1.000	5.5
IL-15	8.50	6–29	17/17	9.00	0–26	33/34	1.000	2.4
IFN-α*	26.24	0–139	16/22	37.90	0–311	37/38	**0.034**	4.3
CCL2	180.89	32–962	23/23	295.22	0–1072	37/38	0.086	1.1
CCL4	16.94	4–137	23/23	33.06	1–229	37/38	0.056	2.4
CCL5	6.30	0–25	16/17	6.30	0–39	33/34	1.000	1.8
CCL11	4.90	0–10	12/17	5.50	0–17	29/34	1.000	2.5
CXCL8	3.08	1–20	22/23	5.58	0–81	36/38	0.060	1.0
CXCL9*	62.20	0–3603	18/20	214.97	21–1351	38/38	**0.006**	1.2
CXCL10	56.27	0–5038	20/22	147.93	0–4677	37/38	0.290	6.1
IL-7*	1.09	0–15	10/23	1.92	0–4	31/38	0.728	1.1
IL-9*	5.50	0–11	15/17	7.57	0–17	33/34	**0.032**	2.5
GM-CSF*	135.45	0–766	18/23	164.05	0–6815	37/38	0.301	2.2
PDGF-BB	3.11	0–14	11/17	2.26	0–9	27/34	1.000	2.9
Basic-FGF	10.62	7–44	17/17	11.39	0–35	33/34	1.000	1.9
VEGF	43.73	22–124	17/17	54.35	0–151	33/34	1.000	3.1

The aqueous concentrations of IFN-γ, IL-10, IL-12, IFN-α, CXCL9 and IL-9 were significantly elevated in the glaucoma group compared to the cataract group (significant p-values are highlighted in bold). ^a^ p-values were obtained from Mann–Whitney U test with Bonferroni correction. *p<0.05 for the comparison of the percentage of measurable samples between glaucoma and cataract groups using Pearson’s χ^2^ analysis. All concentrations of cytokines are in pg/ml.

**Figure 1:**
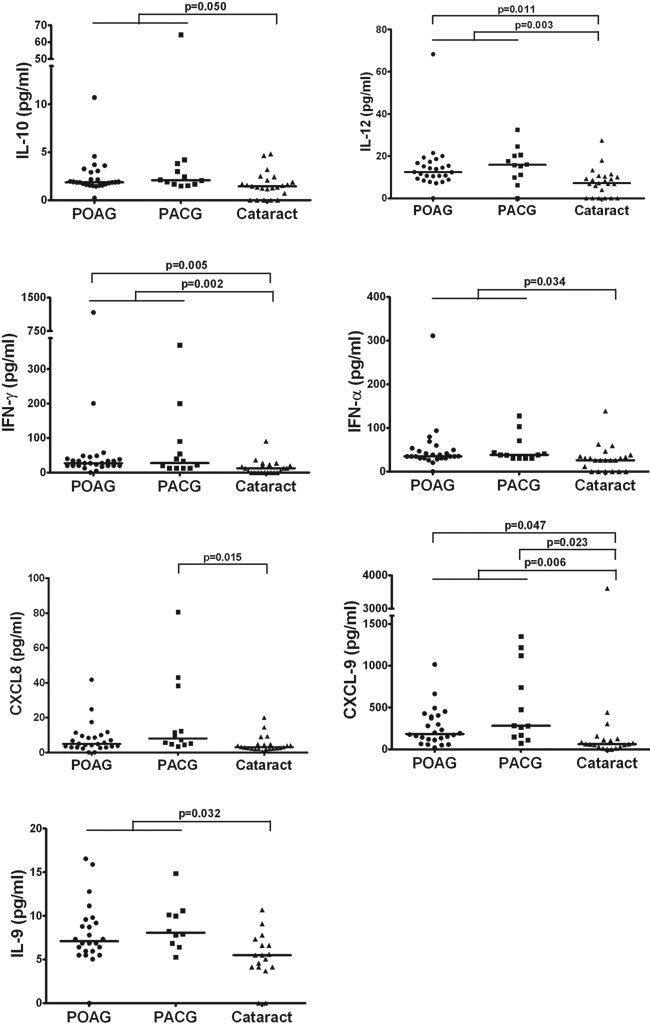
Scatter plots showing distribution level of IL-10, IL-12, IFN-γ, IFN-α, CXCL8, CXCL-9, and IL-9 in aqueous humor from POAG (circles), PACG (squares) and cataract controls (triangles). A Mann–Whitney U test with Bonferroni correction was performed between groups and a significant difference was accepted at p≤0.05. The solid lines indicate median concentrations. Median concentrations of IL-10 (p=0.050), IL-12 (p=0.003), IFN-γ (p=0.002), IFN-α (p=0.034), IL-9 (p=0.032), and CXCL9 (p=0.006) were significantly higher in the glaucoma group compared to the cataract controls. Significantly elevated concentrations of IL-12 (p=0.011), IFN-γ (p=0.005), and CXCL9 (p=0.047) was observed in the POAG aqueous, while CXCL8 (p=0.015) and CXCL9 (p=0.023) concentrations were significantly higher in the PACG group, compared to the cataract group.

Compared to the cataract group, there was a significantly higher proportion of samples with measurable concentrations of the following cytokines in the glaucoma group: IL-2 (p=0.008), IL-6 (p=0.014), IL-10 (p=0.015), IL-12 (p=0.020), IFN-γ (p=0.000), IFN-α (p=0.002), IL-1β (p=0.019), IL-7 (p=0.002), GM-CSF (p=0.015), IL-9 (p=0.046), and CXCL9 (p=0.003; χ^2^ test). Among these cytokines, the median concentrations of IL-10 (p=0.050), IL-12 (p=0.003), IFN-γ (p=0.002), IFN-α (p=0.034), IL-9 (p=0.032), and CXCL9 (p=0.006) were also significantly higher in the glaucoma group (Figure 1).

When the glaucoma group was further separated into POAG and PACG, we found significantly elevated concentrations of IL-12 (p=0.011), IFN-γ (p=0.005), and CXCL9 (p=0.047) in the POAG aqueous compared to the cataract group. Both CXCL8 (p=0.015) and CXCL9 (p=0.023) concentrations were significantly higher in the PACG group compared to the cataract group, after adjustment for differences in axial length using binary logistic regression analysis.

There was no significant difference in any of the cytokine concentration between the two glaucoma subgroups, with and without adjustments for axial length (Table 3).

**Table 3:** Aqueous cytokine concentrations in PACG and POAG groups.

** **	**POAG (n=26)**			**PACG (n=12)**			** **
**Cytokines **	**Median**	**Range**	**Measurable**	**Median**	**Range**	**Measurable**	**Sensitivity**
IL-2	3.12	0–27	24/26	2.84	0–5	10/12	1.6
IFN-γ	26.98*	0–1167	24/26	27.62	13–369	12/12	6.4
IL-13	4.24	0–13	23/26	6.39	3–46	12/12	0.7
IL-17	4.15	0–151	16/26	3.30	1–241	6/12	3.3
IL-6	2.85	0–28	16/26	4.70	2–89	11/12	2.6
IL-10	1.87	0–11	25/26	2.10	1–64	12/12	0.3
IL-12	12.42*	0–68	25/26	15.90	0–32	11/12	3.5
IL-1β	0.72	0–5	23/26	0.72	0–2	10/12	0.6
TNF-α	12.14	0–59	24/26	13.17	5–82	11/12	6.0
IL-1Ra	8.58	0–52	18/24	14.25	3–84	9/10	5.5
IL-15	8.37	0–26	23/24	9.63	5–18	10/10	2.4
IFN-α	35.10	0–311	25/26	38.25	31–128	12/12	4.3
CCL2	287.14	0–615	25/26	325.61	145–1072	12/12	1.1
CCL4	31.84	1–111	25/26	36.88	12–229	12/12	2.4
CCL5	6.30	0–39	23/24	5.52	5–20	10/10	1.8
CCL11	5.30	0–17	20/24	5.89	0–10	9/10	2.5
CXCL8	4.88	0–42	24/26	8.00^†^	3–81	12/12	1.0
CXCL9	183.43*	21–1015	26/26	281.61^†^	73–1351	12/12	1.2
CXCL10	116.80	0–2311	25/26	177.39	31–4677	12/12	6.1
IL-7	1.88	0–3	23/26	2.06	0–4	8/12	1.1
IL-9	7.11	0–17	23/24	8.09	5–15	10/10	2.5
GM-CSF	151.38	0–3139	25/26	185.12	121–6815	12/12	2.2
PDGF-BB	2.10	0–9	9/24	2.69	1–6	5/10	2.9
Basic-FGF	11.39	0–34	23/24	12.16	9–35	10/10	1.9
VEGF	51.44	0–87	23/24	69.6950	27–151	10/10	3.1

*p<0.05, between POAG and cataract; ^†^p<0.05, between PACG and cataract. p-values were obtained from Mann–Whitney U with Bonferroni correction. All concentrations of cytokines are in pg/ml.

### Correlation analysis

There was no correlation between the aqueous cytokine levels and clinical parameters such as preoperative IOP and duration of glaucoma. However, duration of Timolol and Alphagan therapy were found to correlate negatively with CXCL8 (r=-0.588, p=0.035, n=13), respectively. No correlation between age and cytokine concentration was found in the cataract group.

### Classification analysis

Heat map analysis identified the combination of IL-12 and CXCL8 as markers to separate the glaucoma from the cataract group, with an accuracy of 84% for the cataract and 83% for the glaucoma groups. This was confirmed by a decision tree analysis. Based on the decision tree analysis, aqueous humor samples with an IL-12 concentration greater than 11 pg/ml or those with an IL-12 concentration in the range between 7 to 11 pg/ml and a CXCL8 concentration greater than 3.3 pg/ml were categorized as glaucomatous, while the rest were non-glaucomatous. The accuracy of this mathematical formula in separating glaucoma and non-glaucoma cataract samples was the same as the heat map analysis. Using the same analysis, POAG and PACG groups were separated by the combination of IL-13 and CXCL8 with an accuracy of 71% for both groups. The decision tree analysis predicted that aqueous humor samples with IL-13 concentration greater than 6 pg/ml and CXCL8 concentration greater than 8.5 were PACG eyes. In addition, aqueous humor samples with CXCL8 concentration in the range between 4.3 and 8.4 pg/ml and IL-13 concentration less than 4.5 pg/ml were also that from PACG eyes. The accuracy of this mathematical formula in separating PACG and POAG eyes was 71% and 92%, respectively.

## Discussion

This study revealed significant differences in the aqueous cytokine profile of glaucomatous eyes compared to age-matched non-glaucomatous cataract eyes. A total of 6 cytokines were found significantly elevated in glaucoma samples compared to controls, which include IL-10, IL-12, IFN-γ, IFN-α, IL-9, and CXCL-9. The POAG aqueous had a significant increase in IFN-γ, IL-12, and CXCL9 concentrations, thus suggesting a Th1-type proinflammatory response, while the PACG aqueous only had elevated levels of CXCL-8 and CXCL-9 when compared to the cataract controls. The heat map and decision tree analysis showed that both IL-12 and CXCL8 were signature cytokines for differentiating the glaucoma group from the controls and this was consistent with the univariate analysis. To the best of our knowledge, this study is the first comprehensive evaluation of an extensive array of cytokines in the aqueous humor of glaucomatous eyes, both overall as well as between the PACG and POAG subgroups in an Asian population.

Previous studies have reported elevated aqueous concentrations of IL-6 [11,12], TNF-α [6], CXCL8 [11,13], and VEGF [10,17] in glaucomatous eyes. The concentrations of these cytokines were also found to be higher in the glaucoma group compared to the controls in our study. However these differences were not significant after application of the Bonferroni correction. Our study also showed a significantly higher proportion of glaucoma aqueous with a measurable concentration of IL-6 cytokine. We believe that these changes in aqueous cytokines are related to glaucoma and the discrepancies observed in various studies are largely due the sample size and variation within each group. Interestingly, in those patients who had received Alphagan (n=12) and Timolol therapy (n=38), we found a significant negative correlation between the duration of therapy and CXCL8 concentration, thus suggesting that long-term anti-glaucoma treatment may have an effect on aqueous cytokine composition.

The differences in cytokines between glaucomatous and non-glaucomatous aqueous suggest possible complex changes associated with the glaucoma disease process. Increased levels of IL-10, IL-12, and IFN-α are likely associated with activation of leukocytes such as monocytes, macrophages and dendritic cells, in glaucomatous eyes. IL-12 is a key cytokine that links the innate immune response to a Th1 dominant adaptive immune response, by stimulating IFN-γ production from T cells and natural killer cells. CXCL9 is also known to stimulate Th1 type immune responses by inducing the recruitment of activated Th1 cells [18,19]. A positive correlation was found between IL-12 and IFN-γ levels in our study (r=0.356, p=0.028). The Th1 type inflammatory response was evident in the POAG aqueous. However, PACG aqueous showed a less proinflammatory cytokine profile compared to POAG, with only CXCL8 and CXCL9 levels were significantly higher than cataract controls.

IL-9 was found significantly increased in glaucomatous aqueous compared to controls, although this difference was not observed when the glaucoma groups was analyzed separately as POAG and PACG subgroups. This was most likely due to a reduced sample size. IL-9 was measured in almost all glaucomatous and non-glaucomatous aqueous samples tested, thus indicating that it is an important component of the aqueous immune milieu. Originally linked to Th2 cells, IL-9 is expressed by a specific subset of T cells called Th9 cells [20]. Although the main target of IL-9 is the mast cell, it also stimulates the expansion of Th17 and Treg cells [20]. Elevated IL-9 concentration was also observed in idiopathic uveitic aqueous (unpublished data). However, IL-17 level was not significantly elevated in either POAG or PACG groups compared to cataract. The significance of IL-9 in both glaucomatous and non-glaucomatous aqueous remains unknown.

In a study involving the simultaneous analysis of multiple factors from each sample, several factors such as sample size and method of data analysis, can affect the outcome. The Bonferroni correction was applied in this study to determine the significance of changes in cytokine levels. This is a conservative statistical adjustment used for the verification of data observations but it may mask potential changes in exploratory studies. In fact, more cytokines were found to be significantly increased in both POAG and PACG groups compared to controls when the Mann–Whitney U test was performed without Bonferroni correction and these included IL-6, TNF-α, VEGF, and CXCL8 as discussed above. Due to the complicated intrinsic dependence of cytokines, binary logistic multivariate analysis was not able to identify cytokines which could be primarily associated with each type of glaucoma. The classification analysis was employed to compliment the data analysis. Although the present study tried to present data in a conservative way, further studies with a larger sample size would be needed to obtain a confirmative aqueous cytokine profile in both glaucoma and cataract groups.

Furthermore, other parameters such as axial length of the eye, age and gender, may also affect the cytokine data measured [21]. There was no statistically significant difference in age and gender distribution among the study groups in this study. However, we observed a significantly shorter axial length in the PACG group compared to both POAG and cataract groups. Multivariate analysis was performed taking these factors into consideration and the outcome was largely the same as per univariate analysis. Similarly, we did not find any significant correlation between cytokine concentrations and age and axial length in the cataract group.

Lastly, both systemic and topical medications may influence the aqueous immune milieu and they include alpha-agonist prostatic medications and topical anti-glaucoma medications. We were unable to account for the effect of the former therapy as this information was not obtained during the study. Chronic use of topical anti-glaucoma medications is associated with conjunctival and aqueous inflammation [22-24], with the latter shown to be associated with medications such as prostaglandin analogs and alpha-agonists. Topical prostaglandin analogs are commonly used for IOP control and the majority of these eyes were on at least 1 topical medication. As such, this could be a confounder in the interpretation of our results and ideally, aqueous samples obtained from newly diagnosed glaucoma patients before commencement of medical therapy would help determine the significance and role of chronic anti-glaucoma medication on the detection of aqueous inflammation. However, this is not without ethical implications.

In conclusion, the present study showed that primary glaucoma is associated with changes in the aqueous cytokine profile. POAG is different to PACG, with a Th1 type response being observed in the former group, while the latter group had a milder inflammatory response. Further studies will be necessary to determine the significance in the differential inflammatory protein expression found between the two primary glaucoma subgroups as well as to identify the inflammatory protein molecules which may play a role in post-operative wound healing response following filtration surgery.
